# Effect of laparoscopic complete mesocolic excision combined with immunotherapy and its impact on immune function and tumor markers in elderly patients with colon cancer

**DOI:** 10.12669/pjms.39.5.7090

**Published:** 2023

**Authors:** Tao Zhang, Qian Lin, Zhi Liu, Hua Yang

**Affiliations:** 1 Tao Zhang, Department of General Surgery, The No.2, Hospital of Baoding, Baoding 071051, Hebei, P.R. China; 2 Qian Lin, Department of Nursing, The No.2, Hospital of Baoding, Baoding 071051, Hebei, P.R. China; 3 Zhi Liu, Department of General Surgery, The No.2, Hospital of Baoding, Baoding 071051, Hebei, P.R. China; 4 Hua Yang, Department of Nursing, The No.2, Hospital of Baoding, Baoding 071051, Hebei, P.R. China

**Keywords:** Laparoscopic complete mesocolic excision, Immunotherapy, Colon cancer in the elderly, Immune function, Tumor markers

## Abstract

**Objective:**

To determine the effect of laparoscopic complete mesocolic excision combined with immunotherapy and its impact on immune function and tumor markers in elderly patients with colon cancer.

**Methods:**

This is a clinical comparative study. Eighty elderly patients with colon cancer hospitalized in the No.2 Hospital of Baoding from May 2020 to May 2022 were randomly divided into two groups, with 40 cases in each group. Patients in the study group received laparoscopic complete mesocolic resection combined with ubenimex orally. While patients in the control group received routine open surgery. The surgical indexes, surgical complications, and the changes of immune molecules and tumor markers before and after treatment were compared between the two groups.

**Results:**

The amount of intraoperative bleeding, retention time of drainage tube and postoperative length of stay in the hospital in the study group were significantly better than those in the control group (p=0.000). The incision length of the study group was significantly shorter than that of the control group, the number of lymph nodes removed during the operation was significantly higher than that of the control group, and the incidence of surgical complications was significantly lower than that of the control group (p<0.05). After treatment, the levels of immune molecules in the study group were remarkably higher than those in the control group (p<0.05), while the levels of tumor markers were much lower than those in the latter group (p=0.000).

**Conclusion:**

Laparoscopic complete mesocolic excision combined with immunotherapy exhibits a superior therapeutic effect to traditional open surgery in elderly patients with colon cancer, and is worthy of clinical promotion.

## INTRODUCTION

With the changes in living and dietary habits of the public, colon cancer has become the second most common cancer in the world, with a gradual increase in its incidence.^1^,^2^ Surgery is still the primary approach for the treatment of colon cancer.^3^ Mesocolic excision has gradually become the standard radical operation for colon cancer, which can significantly improve the prognosis of colon cancer patients.^4^ At present, laparoscopic-assisted surgery has been widely adopted for colon cancer.^5^ Laparoscopic complete mesocolic excision has a larger surgical margin than that of traditional radical surgery.

Besides, due to its high difficulty of detection and diagnosis in the early stage, these patients may lose the best opportunity for surgery and experience significantly compromised therapeutic effects. Meanwhile, elderly patients have low immunity, slow postoperative recovery, and high risk of serious surgical complications than young patients.^6^ At the same time, there are few studies on the effect and safety of laparoscopic complete mesocolic excision combined with postoperative immunotherapy in elderly patients. Accordingly, the present study was performed to evaluate the effect of laparoscopic complete mesocolic excision combined with immunotherapy and its impact on immune function and tumor markers in elderly patients with colon cancer.

## METHODS

This is a clinical comparative study. Eighty elderly patients with colon cancer hospitalized in the No.2 Hospital of Baoding from May 2020 to May 2022 were randomly divided into two groups, with 40 cases in each group. They’re existed comparability between the two groups as there was no significant difference in the comparison of general data between groups (Table-I). This study has been approved by the medical ethics committee of Ethical Approval: The No.2 Hospital of Baoding (No.:2041ZF014; date: March 20, 2021), and written informed consent was obtained from all participants.

**Table-I:** Comparison of general data between the two groups (*χ̅*±*S*) n=40.

	Study group	Control group	t/χ^2^	p
Male (n %)	23 (57.50%)	25 (62.50%)	0.208	0.648
Age (years)	69.83±3.17	69.53±3.42	0.407	0.685
BMI (kg/m^2^)	34.32±4.34	34.09±3.54	0.257	0.798
Course of disease (years)	1.78±0.70	1.68±0.73	0.626	0.533
***Tumor site***			0.474	0.491
Left	14 (35.00%)	17 (42.50%)		
Right	26 (65.00%)	23 (57.50%)		
***Tumor stage***				
I	8 (20.00%)	9 (22.50%)	0.075	0.785
II	14 (35.00%)	15 (37.50%)	0.054	0.816
III	18 (45.00%)	16 (40.00%)	0.205	0.651
ECOG score	0.3±0.54	0.68±0.66	0.372	0.711

p>0.05.

### Inclusion criteria:

- Patients aged 65-75 years old.
- Patients who met the diagnostic criteria of colon cancer and had surgical indications.^7^
- Patients with complete relevant data.
- Patients who agreed to be included in the study and provided the written informed consent and those agreed by family members.
- Patients who agreed to be followed up.

### Exclusion criteria:

- Patients with severe mental disorders who cannot cooperate to complete the study.
- Patients with other serious underlying diseases that cannot be corrected and cannot tolerate surgery.
- Patients with severe infectious diseases.
- Patients with other malignant tumors.
- Patients with an estimated survival time of <six months.
- Patients with previous abdominal surgery.
- Patients with distant metastasis of tumor.

Patients in the study group received laparoscopic complete mesocolic excision combined with immunotherapy. Via tracheal intubation under general anesthesia, the operation was started through a medial approach by using the four-hole method. After that, the ileocolic blood vessels, the gastrocolonic venous trunk and the branches of the middle colonic artery were severed from the root of the blood vessels, after which the mesenteric lymph nodes were dissected. Further sharp dissection was made along the visceral layer of fascia around the mesocolon and the avascular area between spaces to completely remove the tumor, blood vessels and visceral fascia around lymph nodes.

For left colon cancer, a sharp dissection was carried out for the visceral fascia covering the descending colon and sigmoid colon as well as the parietal fascia covering perirenal fat, ureter, etc. After the recovery of food intake after operation, patients were provided with oral ubenimex (30 mg, once a day). Meanwhile, patients in the control group were given open surgery, with the same surgical margin and operation as those in the study group. Postoperative routine treatment in this group included nutritional supplements, correction of water and electrolyte, supplementation of albumin, etc.

### Observation indicators:

**(1)** Surgical indicators were compared and analyzed between the two groups, including average operation time, total amount of intraoperative bleeding, extraction time of drainage tube, postoperative length of stay in the hospital. **(2)** The number of dissected lymph nodes intraoperatively was compared and analyzed between the two groups. **(3)** Occurrence of surgical complications was also compared between the study group and the control group. **(4)** As for the comparative analysis of immune molecules and tumor markers, venous blood was collected from each group before operation and three months after operation. Further detection was performed focusing on immune molecules of CD3^+^, CD4^+^, CD8^+^ and CD4^+^/CD8^+^ and tumor markers of serum colon cancer-specific antigen 2 (CCSA-2), pleiotropic growth factor (pleiotrophin, PTN) and soluble interleukin two (SIL2) to compare and analyze the changes of these indicators before and after treatment. The maximum follow-up time for patients in both groups was three months. And case data collection ceased in May 2022.

### Statistical analysis:

All data were statistically analyzed using SPSS 20.0 software. The measurement data were presented in the form of (*X̅*±*S*). Two independent samples t-test and paired t-test were respectively used for inter- and intra-group analyses. The comparison of rate adopted c^2^ test. P<0.05 was used to indicate the existence of a statistically significant difference.

## RESULTS

As shown in Table-II, the study group had significantly less amount of intraoperative bleeding, as well as a shorter retention time of drainage tube and postoperative length of stay in the hospital than those in the control group (p=0.000). The incision length was obviously shorter in the study group than that in the control group (p=0.000).

**Table-II:** Comparison of surgical indicators between the two groups (*χ̅*±*S*) n=40.

Groups	Operation time (min)	Amount of bleeding (ml)*	Retention time of drainage tube (d)*	Postoperative length of stay (d)*	Incision length (cm)*
Study group	161.38±8.51	86.50±5.57	3.65±0.80	7.75±1.26	6.30±0.56
Control group	158.60±5.29	110.85±3.61	4.85±0.77	12.83±1.15	11.15±0.48
t	1.752	23.209	6.827	18.834	41.313
p	0.084	0.000	0.000	0.000	0.000

* p<0.05.

The comparative analysis of the difference in the number of dissected lymph nodes intraoperatively between the two groups suggests that it was significantly higher in the study group than that in the control group, with consistent results observed based on the subgroup analyses according to different tumor stages and tumor sites (p<0.05, Table-III). According to the comparative analysis, the incidence of surgical complications in the study group was much lower than that in the control group, and the difference was statistically significant (p=0.032; Table-IV).After treatment, the levels of CD3^+^, CD4^+^ and CD4^+^/CD8^+^ were obviously higher in the study group than those in the control group (p<0.05, Table-V).After treatment, the levels of CCSA-2, PTN and SIL2 were obviously lower in the study group when compared with those in the control group (p=0.000, Table-VI).

**Table-III:** Comparison of the number of dissected lymph nodes intraoperatively between the two groups (*χ̅*±*S*) n=40.

Groups	Stage I*	Stage II*	Stage III*	Left colon*	Right colon*
Study group	15.88±2.37	16.50±1.84	19.85±2.48	18.48±1.69	22.05±1.34
Control group	14.65±1.99	14.63±1.33	15.75±2.10	16.95±1.72	18.43±2.54
t	2.504	5.219	7.992	3.990	7.983
p	0.014	0.000	0.000	0.000	0.000

* p<0.05.

**Table-IV:** Comparison of the incidence of surgical complications between the two groups (*χ̅*±*S*) n=40.

Groups	Incision infection	Lung infection	Venous thrombosis of lower limbs	Lymphorrhagia	Intestinal obstruction	Poor wound healing	Incidence rate (%)*
Study group	0	1	1	1	2	0	5 (12.50%)
Control group	3	0	3	1	4	2	13 (32.50%)
c^2^							4.588
p							0.032

* p<0.05.

**Table-V:** Comparison of T lymphocyte subset level in two groups before and after treatment (*χ̅*±*S*) n=40.

Indicators		Study group	Control group	t	p
CD3^+^ (%)	Before treatment	43.03±5.49	42.97±6.22	0.048	0.962
	After treatment*	47.80±6.37	44.13±6.15	2.621	0.011
CD4^+^ (%)	Before treatment	27.28±4.09	27.01±3.83	0.302	0.764
	After treatment*	35.68±4.64	31.22±4.76	4.243	0.000
CD8^+^ (%)	Before treatment	21.62±3.70	21.08±3.70	0.653	0.516
	After treatment	22.25±3.73	22.32±3.90	0.082	0.935
CD4^+^/CD8^+^	Before treatment	1.27±0.10	1.29±0.07	1.149	0.254
	After treatment*	1.62±0.16	1.41±0.15	5.992	0.000

* p <0.05.

**Table-VI:** Comparison of tumor marker levels between the two groups before and after treatment (*χ̅*±*S*) n=40.

Indicators		Study group	Control group	t	p
CCSA-2 (mg/L)	Before treatment	122.48±7.53	119.89±7.47	1.545	0.126
	After treatment*	62.74±8.14	73.65±7.26	6.323	0.000
PTN (ng/L)	Before treatment	183.65±7.24	180.55±7.44	1.887	0.063
	After treatment*	112.06±5.31	139.81±5.72	22.480	0.000
SIL2 (U/L)	Before treatment	2.27±0.76	2.30±0.42	0.218	0.828
	After treatment*	1.12±0.27	1.59±0.35	6.653	0.000

* p <0.05.

## DISCUSSION

In our study, the number of dissected lymph nodes intraoperatively in the study group was significantly higher than that in the control group, regardless of different tumor stages or tumor sites (p=0.00), which may be attributed to the amplification effect of laparoscopy. Meanwhile, the study group was discovered to have a smaller amount of intraoperative bleeding, as well as a shorter retention time of drainage tube, length of stay in the hospital postoperatively and postoperative incision length. In addition, the incidence of complications in the study group was obviously lower than that in the control group.

It has been recognized that there is an intimate association between the levels of postoperative tumor markers and the risk of tumor recurrence.^8^ SIL2 can inhibit the immune function of the body; PTN is a pleiotropic growth factor and has a variety of biological functions that can cause tumor cell metastasis and proliferation.^9^ CCSA-2 plays an important role in evaluating the prognosis of diseases, which is a colon cancer-specific molecule.^10^ In the present study, the levels of CCSA-2, PTN and SIL2 in the study group were lower than those in the control group after treatment. These data further reveal that compared with open radical surgery, laparoscopic surgery can remove tumor tissues more thoroughly, and also has a lower risk of postoperative recurrence.

Colon cancer is featured by relatively higher clinical morbidity and mortality, and high incidence in elderly men.^11^ Surgery has been accepted clinically as the major approach to treating colon cancer.^12^ Through the resection of the tumor primarily, the traditional radical resection of colon cancer may cause the extrusion and then the spread of tumor tissues during intraoperative separation and resection, leading to a greater risk of postoperative recurrence.^13^ Significantly, complete mesocolic excision is a novel therapeutic option surgically, with significant clinical effects.^14^ With the assistance of a laparoscope, it may provide a clear field of vision for a surgical operation with minimal damage to the patients, exhibiting advantages of less intraoperative bleeding, short hospital stay, low complications, etc.^15^

Previous research^16^ has reported that the number of dissected lymph nodes during colon cancer surgery was an independent factor affecting the clinical prognosis of patients with colon cancer. The use of a laparoscope can magnify the field of vision to display the local anatomical visual field more clearly, which can facilitate the identification of blood vessels to assist in the dissociation of the root of blood vessels and dissection of lymph nodes, eventually protecting the surrounding adjacent tissue structures, and reducing secondary damages of surgery.

Enhancement of immunity and body resistance is of great significance for the postoperative rehabilitation of elderly patients.^17^ Ubenimex is a new generation of immunopotentiator that can enhance immune function, which can can be used cooperatively or jointly for the treatment of patients with various solid tumors.^18^ Yang et al. suggested that ubenimex also had a certain direct antitumor effect.^19^ At the same time, ubenimex, an inhibitor of CD13, can be used as an immune adjuvant to improve the immune state of patients.^20^ Similarly, in our study, after treatment, the levels of CD3^+^, CD4^+^ and CD4^+^/CD8^+^ were obviously elevated in the study group than those in the control group (p<0.05), which confirmed significant improvement in the cellular immune function of patients after applying immunotherapy jointly.

### Limitations:

However, the small sample size and the lack of follow-up are two major limitations of our study. Our future research will be continued by including more samples, having follow-ups, and further exploring the impact of the therapeutic scheme on the long-term effect and survival of patients. Through relevant studies, we hope to realize a more comprehensive evaluation of its long-term therapeutic effect so that more patients can benefit from this treatment.

## CONCLUSION

Findings in our study supported that laparoscopic complete mesocolic excision combined with immunotherapy has a superior therapeutic effect to traditional open surgery in elderly patients with colon cancer. Simultaneously, this therapeutic schedule also has a certain effect on improving the cellular immune function of patients, reducing the levels of tumor markers and the incidence of postoperative complications.

### Authors’ Contributions:

**TZ** and **QL:** Carried out the studies, participated in collecting data, and drafted the manuscript, are responsible and accountable for the accuracy and integrity of the work.

**ZL:** Performed the statistical analysis and participated in its design.

**HY:** Participated in acquisition, analysis, or interpretation of data and draft the manuscript.

All authors read and approved the final manuscript.
